# Orphan drug: Development trends and strategies

**DOI:** 10.4103/0975-7406.72128

**Published:** 2010

**Authors:** Aarti Sharma, Abraham Jacob, Manas Tandon, Dushyant Kumar

**Affiliations:** Drug Regulatory Affairs, Getwell Pharmaceuticals, Udyog Vihar, Gurgaon, India

**Keywords:** Exclusivity, incentives, orphan drug, pipeline drugs, rare diseases

## Abstract

The growth of pharma industries has slowed in recent years because of various reasons such as patent expiries, generic competition, drying pipelines, and increasingly stringent regulatory guidelines. Many blockbuster drugs will loose their exclusivity in next 5 years. Therefore, the current economic situation plus the huge generic competition shifted the focus of pharmaceutical companies from the essential medicines to the new business model — niche busters, also called orphan drugs. Orphan drugs may help pharma companies to reduce the impact of revenue loss caused by patent expiries of blockbuster drugs. The new business model of orphan drugs could offer an integrated healthcare solution that enables pharma companies to develop newer areas of therapeutics, diagnosis, treatment, monitoring, and patient support. Incentives for drug development provided by governments, as well as support from the FDA and EU Commission in special protocols, are a further boost for the companies developing orphan drugs. Although there may still be challenges ahead for the pharmaceutical industry, orphan drugs seem to offer the key to recovery and stability within the market. In our study, we have compared the policies and orphan drug incentives worldwide alongwith the challenges faced by the pharmaceutical companies. Recent developments are seen in orphan drug approval, the various drugs in orphan drug pipeline, and the future prospectives for orphan drugs and diseases.

The spiraling cost of drug development in tune with stringent regulations, coupled with the low return on investment, often tends to discourage pharmaceutical innovators from developing products for extremely small patient populations. Rare diseases in small patient populations thus “orphaned” by the pharmaceutical industry, having but a few approved drug treatment options available are called “orphan diseases.”

A medicinal product designated as an orphan drug is one that has been developed specifically to treat a rare medical condition, the condition itself being referred to as “orphan disease.” It may be defined as drugs that are not developed by the pharmaceutical industry for economic reasons but which respond to public health need. Actually, the indications of a drug may also be considered as “orphan” since a substance may be used in the treatment of a frequent disease but may not have been developed for another, more rare indication.

The orphan diseases are often so rare that a physician may observe only one case a year or less. Academic physician-scientists have tried to fill this therapy vacuum by working on developing orphan drugs. But many disincentives are involved, which include career disincentives, lack of funding, and the multiple areas of expertise that are required. Positive developments include formation of the National Organization for Rare Diseases, the Orphan Drug Act, the development of a grant program to fund orphan drug development, the formation of the National Institutes of Health Office of Rare Diseases, and the passage of orphan drug legislation by other countries. Progress has increased, but the 300 orphan drugs and devices approved in the last 25 years are still only a drop in the bucket compared with the many thousands of orphan diseases.[1]

Diseases that manifest in patient populations representing at the maximum 6–8% of the world population are defined as rare diseases or orphan diseases. They are pathologies whose incidence at birth is less than 1 in 2000. Symptoms of some rare diseases may appear at birth or in childhood, including infantile spinal muscular atrophy, lysosomal storage disorders, patent ductus arteriosus (PDA), familial adenomatous polyposis (FAP), and cystic fibrosis. However, more than 50% of rare diseases appear during adulthood, such as renal cell carcinoma, glioma, and acute myeloid leukemia. Today there are over 5000 rare diseases listed in the world with five new rare diseases being described every week in the medical literature. And 80% of rare diseases have been identified to genetic origins. Other rare diseases are the result of infections (bacterial or viral) and allergies, or are due to degenerative and proliferative causes.[2]

The assignment of orphan status to a disease and to any drugs developed to treat it is a matter of public policy in many countries and has resulted in medical breakthroughs that may not have otherwise been achieved due to the economics of drug research and development. Also if it is intended for the diagnosis, prevention, or treatment of a life-threatening, seriously debilitating, or serious or chronic condition and without assured incentives, it is unlikely that expected sales of the medicinal product would cover up for the investment in the development; and since no satisfactory method of diagnosis, prevention, or treatment of the condition concerned is authorized, or, if such method exists, the medicinal product will be of significant benefit to those affected by the condition.[3] It is illustrated in Table 1.

**Table 1:** Classification of orphan drugs

Type	Expected profits	Available medication
Little/no commercial benefit	Poor	Inadequate
Commercial benefit	Good to excellent	Inadequate
For rare disease that can currently be treated	Variable	Adequate
Unprofitable for a common disease	Poor	Inadequate
Orphan for both rare and common disease	Variable	Variable

Hence orphan drugs can be defined as “those drugs intended to treat either a rare disease or a more common disease where manufacturer cannot expect to make profits.” For example, drugs and vaccine for tropical diseases are also defined as orphan drugs because patient sufferings from these diseases, although numbering tens of millions, are too poor to pay the price of medications.[4]

## ‘Orphan Drug’ Status: The Various Probabilities

- *Products intended to treat rare diseases*. These products are developed to treat patients suffering from very serious diseases for which no treatment, or at least a satisfactory one, has so far been available. These diseases affect only a small proportion of the population most often at birth or in infancy. The number of rare diseases for which no treatment is currently available is estimated to be between 4,000 and 5,000 worldwide.
- *Products withdrawn from the market for economic or therapeutic reasons*. Few drugs which are withdrawn from the market for some reasons, e.g., thalidomide widely used as a hypnotic drug some years ago for its high teratogenic (triggering fetal malformations) risk may show a very interesting therapeutic application, i.e., analgesic proprieties in rare diseases such as leprosy and lupus erythematosus. These are diseases for which no satisfactory treatment is available.
- The global orphan drugs market reached $84.9 billion in 2009 growing from $58.7 billion in 2006 from $54.5 billion in 2005. The market is expected to grow at a compound annual growth rate (CAGR) of nearly 6% to reach $112.1 billion by 2014. The U.S. accounted for 51% of the market in 2009 and is expected to grow at a CAGR of 8.9% to reach $65.9 billion by 2014.
- Regionally, U.S. revenues in the market grew to $32.5 billion accounting for 55% of the market in 2006. The U.S. market is expected to grow at a CAGR of 8% to reach $47.8 billion by 2011.
- Biological drugs account for a major share (64.3%) of the orphan drug market with sales of $54.6 billion in 2009 as compared to $35.3 billion in 2006 and $30.2 billion in 2005. The size of the biological orphan drug market is projected to grow at a 6.9% CAGR to reach $76.2 billion by 2014 illustrated in Table 2.
- Orphan drugs for the cancer sector generated the largest amount of revenues, $30.6 billion in 2009, and accounting for 36% of the market. Revenues for cancer-related orphan drugs are expected to grow at a CAGR of 10% to reach $49.7 billion in 2014.[6,7]

**Table 2:** Global orphan drugs demand by value, 2005–2011 ($, million)

	2005	2006	2011	CAGR (%) 2006–2011
Biologics	30,200	35,300	53,400	9
Nonbiologics	24,300	23,400	28,400	4
Total	54,500	58,700	81,800	7

## Defining of ‘Orphan Drugs’: Beyond National Boundaries

### United states

As defined in the United States, any drug developed under the Orphan Drug Act of January 1983 (ODA) is an orphan drug. The ODA is a federal law concerning rare diseases (orphan diseases) that affect fewer than 200,000 people in the United States or are of low prevalence (less than 5 per 10,000 in the community).[8–11]

### Europe

A disease or disorder that affects fewer than 5 in 10,000 citizens is the definition for rare in Europe (Orphan Drug Regulation 141/2000). At first glance, this may seem a small number, but by this definition, rare diseases can affect as many as 30 million European Union citizens. According to EURORDIS (European Organization for Rare Diseases), the number of rare diseases numbers from about 6,000 to 8,000, most of which have identified genetic conditions, with medical literature describing approximately five new rare conditions every week. Twenty-five to Thirty million people are reported to be affected by these diseases in Europe.[12,13]

### Japan

A drug must meet the following three conditions in order to be considered for orphan drug designation in Japan. Any disease with fewer than 50,000 prevalent cases (0.4%) is Japan’s definition of rare. The drug treats a disease or condition for which there are no other treatments available in Japan or the proposed drug is clinically superior to drugs already available on the Japanese market. The applicant should have a clear product development plan and scientific rationale to support the necessity of the drug in Japan. Once clinical trials are completed, a New Drug Application (NDA) can be submitted. It is important to keep in mind that while Japan has orphan drug legislation, this legislation has room for interpretation. The MHLW makes orphan drug designation and approval decisions on a case-by-case basis. This is especially true when determining the number of Japanese clinical trials required for approval.

Few of the recently approved orphan drugs include Glaxo’s Lexiva (Fosamprenavir) for HIV infection, Genzyme’s Fabrazyme (Agalsidase beta) for Fabry disease, and Novartis’s Visudyen (Verteporfin) for age-related macular degeneration.[14]

### Australia

The Therapeutic Substances Regulations does not define a rare disease or orphan indication in terms of the number of patients, but rather indicates that it must not be intended for use in more than 2000 patients a year if it is a vaccine or *in vivo* diagnostic. In order to attain the orphan designation, “the application must show why the medicine is an orphan drug.” In Australia, orphan drugs are drugs used to treat diseases or conditions affecting fewer than 2,000 individuals at any one time (0.2%).[15]

### Canada

Canada has no official “orphan disease” status; however, based on international standards, it could be defined as diseases with a potential patient population numbering between 3,300 (Australian standards) to 22,500 (US definition).[16]

## The Asian Perspective to ‘Orphan Drugs’

### India

The need for such an act is thus evident from the initiative by the Indian Pharmacists and the Government to implement Laws, which would strengthen the health infrastructure and provide relief to the numerous rare disease sufferers throughout the country. A group of pharmacologists at a conference held by the Indian Drugs Manufactures Association in 2001 requested the Indian Government to institute the Orphan Drug Act in India.[17]

### Taiwan

The official definition of rare disorders is a disease if it is prevalent in 1:10,000 people. On February 9, 2000, Taiwan’s Legislative Yuan implemented the Rare Disease and Orphan Drug Act to improve the diagnosis, treatment, and prevention of rare diseases in Taiwan. In particular, the Act aims to provide patients with easier access to pharmaceuticals for the treatment of rare diseases by promoting the supply, manufacturing, and R and D of these products. To carry out the Act, the Department of Health (DOH) established the Committee for the Review and Examination of Rare Diseases and Orphan Drugs.

By the end of April of 2006, the government classified 159 diseases to be under the rare disease category, thereby protecting patients under the “Rare Disease Control and Orphan Drug Act” with 2,117 cases confirmed. TFRD (Taiwan Foundation for Rare Disorders) has been serving patients with 191 rare diseases affecting 2,252 people. So far 77 orphan drugs and 40 special nutrients have been approved for treating patients by the government. According to the act, these drugs and special nutritional food can be imported and the National Health Insurance reimburses up to 56 out of 77 orphan drugs.

The health standards in Taiwan are among the best in Asia, and the country boasts high life expectancy - about 75 years for men and 80 for women. As of 2005, Taiwan had over of 20,000 medical care institutions, with over 50 hospital beds for every 10,000 citizens. The patient-to-doctor ratio was approximately 750 to 1 in 2004. Taiwan’s DOH is responsible for ensuring the availability and efficiency of medical treatment in Taiwan. Taiwan’s pharmaceutical market is currently valued at around $2.75 billion, and the government is continuing to improve drug regulations and standards in order to attract more foreign enterprises and investments. In early 2005, the amount of Western drug imports to Taiwan had risen over 100% since 2004 to New Taiwan Dollar (NT) 1.45 billion (US $44 million), according to the Ministry of Economic Affairs.

### Korea

For orphan drug designation in Korea, less than 20,000 people in Korea suffer from the disease/condition, or there is no available treatment for the disease/condition in Korea. If the product is manufactured in Korea, the total production should be less than 5 billion won (U.S. $5 million). If the drug is manufactured outside of Korea and imported for sale, the total imports of the drug should be less than U.S. $5 million.

The Korean pharmaceutical market is currently valued at around $6.5 billion, the third largest in Asia behind that of Japan and China. While the market has been growing steadily at 7–9% per year for the past several years, the Korea Food and Drug Administration (KFDA) continues to work on the internationalization and improvement of the country’s pharmaceutical regulations.[18]

In Korea, orphan drugs are supplied to patients by pharmaceutical companies or the Korea Orphan Drug Center. As of October 2002, over 130 orphan drugs have been approved by the KFDA. The orphan drug application process takes around 6-9 months to complete. Some of the recently approved orphan drugs in Korea include Abbott’s Kaletra (Lopinavir plus Ritonavir) capsules and solutions for HIV infection and Schering Plough’s Bonefos (Disodium Clodronate) solutions and capsules for the treatment of hypercalcemia and osteolysis due to malignancy.

### Hong Kong

Hong Kong boasts a small but wealthy population and the country’s healthcare standards are among the highest in Asia. The pharmaceutical market is valued at around $1.6 billion and offers advanced technologies and a very high standard of care. While there is some Hong Kong based drug manufacturing, more advanced drugs are generally imported. The DOH is responsible for health legislation and policy in Hong Kong.

An orphan drug applicant may register their drug under the New Chemical Entity (NCE) registration process, which was established for new, life saving drugs. In this case, the application will be processed immediately and reviewed by the Hong Kong DOH Pharmaceutical Licensing Committee. This Committee only meets four times a year, so applicants should make an effort to submit their application several weeks prior to a Committee meeting to reduce processing time.

A second registration process is available for those applicants which cannot meet the NCE application requirements. The second option, registering under the “normal” registration process, takes 6–9 months to complete.

### Singapore

Like Hong Kong, Singapore is small but economically advanced, offering a highly developed healthcare system. The country also serves as an Asian hub for many medical companies. A number of large pharmaceutical companies, such as Pfizer and GlaxoSmithKline, have established their presence in Singapore and continue to expand their manufacturing and research facilities.

Singapore’s Medicines Act regulates pharmaceuticals in the country, specifically mentions orphan drugs, that portion of the law has never been “activated.” Therefore, the definition of an orphan drug is not 100% clear in Singapore and companies can encounter difficulty receiving orphan drug designation. The following information will be supportive in making an orphan drug claim.

- The drug is used in life-threatening conditions and plays a critical role in the management of that condition.
- The disease/condition currently affects a very limited number of patients.
- A rough estimate of the number of patients affected by the disease/condition should be provided.

If the drug meets the above conditions and is designated as an orphan drug by the Ministry of Health, it will be given top priority during the registration process. To date, reimbursement of orphan drugs in Singapore has been very challenging.[19]

The various countries orphan drug policies are illustrated in Table 3.

**Table 3:** Orphan drug regulations in different countries

Country	Description
Australia	No orphan drug policy
	Special access scheme (SAS) for unapproved drugs Provision for reduction of fees under cost recovery for products used to treat rare but clinically significant conditions for which there is only a limited market
Canada	No orphan drug policy
	Emergency drug release program/investigational new drugs (EDRP/IND) provides access to unapproved drugs SR and ED (scientific research and experimental development) tax incentive program would support R and D in the area of orphan drugs Provision for reduction of fees for small market drugs under cost recovery Process patents granted for biotechnology products Conditional approvals proposed under new licensing framework
European Union	Development of an orphan drug policy is part of the 1996 work program
	The policy is likely to include:
	designation based on prevalence of disease in the population of less than 0.05% (about 180,000 patients) *and* no expectation of profitability and subject to review and withdrawal if criteria no longer apply shared cost program to support research in addition to the BIOMED program already in place monitored release program in addition to the current provision which permits marketing authorization for some drugs based on a limited dossier
	Development of a telematic network to facilitate clinical trials and research
	legislation is already in place providing market exclusivity and provisions for fee exemptions under cost recovery
	Many member states already have incentives in place for R and D related to orphan diseases
	Individual member states control access to drugs through their own programs (see France, U.K.)
France	No orphan drug policy but leading initiatives in the E.U.
	Temporary approval (ATU) for “orphan” drugs may be granted based on available data for a time period of 3 months to 1 year
	Approval may apply to a cohort of patients or may require release on an individual patient basis
Japan	Orphan drug program
	Designation granted based on prevalence of disease in the population of less than 0.05% Grant program for R and D for manufacturers and importers of orphan drugs guidance and advice available to industry on both R and D and NDA application procedures Tax incentives granted to manufacturers doing R and D on orphan drugs NDA for orphan drugs are given priority review If drug is marketed, a portion of profits in excess of 100 million yen must be paid to the government
U.K.	No orphan drug policy (see E.U.)
	Historically, some government research funds have been made available for the development of drugs for rare diseases Practitioners can procure unapproved drugs for individual patients based on clinical judgment An application under exceptional circumstances can be made when insufficient information on the safety, quality, and/or efficacy of a product exists or when it may be unethical to collect such information. Manufacturers must detail PMS (post-marketing surveillance) studies to be undertaken Provision for abating fees for NDA (new drug application) for small market drugs under cost recovery
U.S.	Orphan drug act (January 4, 1983)
	Designation granted based on prevalence of disease in the population of less than 200,000 people (approximately 0.1%) or no reasonable expectation of profitability Protocol assistance to design research protocols Tax credits for clinical research Market exclusivity Funding grants for clinical research to support development Penalty for intentionally false statement of orphan status Parallel track program and treatment INDs provide access to unapproved drugs Process patents granted for biotechnology products Accelerated approvals

## The Orphan Drug Stimulus

In the *European Union*, companies with an orphan designation for a medicinal product benefit from incentives by protocol assistance (scientific advice during the product development phase). They are given the marketing authorization for 10-year marketing exclusivity and are given financial incentives in terms of fee reductions or exemptions and national incentives.

In the European Union, since 1 January, 2007, orphan medicinal products are eligible for the following level of fee reductions: 100% reduction for protocol assistance and follow up; 100% reduction for preauthorization inspections; 50% reduction for new applications for marketing authorization; 50% reduction for postauthorization activities, including annual fees (applies only to small and medium sized enterprises), in the first year after grant of a marketing authorization.

*The Food and Drug Administration* has charged The Office of Orphan Products Development (OOPD) to dedicate its mission to promoting the development of products that demonstrate promise for the diagnosis and/or treatment of rare diseases or conditions. It administers the major provisions of the orphan drug act (ODA), which provide incentives for sponsors to develop products for rare diseases. The ODA has been very successful for more than 200 drugs and biological products for rare diseases have been brought to market since 1983. In contrast, the decade prior to 1983 saw fewer than ten such products come to market. In addition, the OOPD administers the Orphan Products Grants Program which provides funding for clinical research in rare diseases. The FDA funds the development of orphan products through its grants program for clinical studies. The Request for Applications (RFA) announcing availability of funds is published in the Federal Register each year – usually in June. Eligibility for grant funding is extended to medical devices and medical foods for which there is no reasonable expectation of development without such assistance. Applications are reviewed by panels of outside experts and are funded by priority score.[20,21]

In Japan, drug companies that are granted orphan drug designation are eligible to receive the following benefits:

The MHLW (Ministry of Health, Labor and Welfare) has a consultation service specifically for orphan drug designation applicants, which is free of charge.

The applicant may receive financial aid from the Japanese government for the collection of supporting data, such as clinical trials, bridging studies, etc. Specifically, the applicant may receive financial aid for as much as 50% of the cost of the clinical trials, as well as tax exemptions of up to 6% of research costs and 10% of corporate tax.

The application will be placed on a fast-track approval process, which generally proceeds much smoother than that of regular drugs. In theory, the fast-track approval process takes 10 months while the approval for regular drugs takes at least 12 months.

Product renewal for orphan drugs is every 10 years, compared to every 6 years for other drugs.

The MHLW determines the amount of clinical data required for an orphan drug application and approval on a case-by-case basis. Of course, Japanese data are most valuable. Japanese data are considered supportive in terms of getting the product approved. Generally, foreign or Asian (non-Japanese) data are considered more as reference data by the MHLW. In Japan, as in other Asian countries, it is particularly important to identify doctors or key opinion leaders (KOLs) who may be interested in your orphan drug. In order to obtain the strongest support for your product, it is best to target doctors focused on the specific disease/condition your drug treats. Backing from relevant Japanese associations can also be very important.

In Taiwan, pharmaceuticals designated as orphan drugs are not required to undergo clinical trials for approval in Taiwan as long as the drug has already been approved by the US FDA. If however the drug has not received US FDA approval, local clinical trials in Taiwan will be required. The application must be submitted by a subsidiary of the manufacturer who has an office in Taiwan or by a local Taiwanese agent (local distributor, local company office, or independent third party). The DOH’s review process takes 6-10 months to complete. The ability for patients to quickly obtain medication for a rare disease is still an issue in Taiwan. Some conditions are officially classified as “rare diseases” under the Rare Disease Prevention and Medicine Law in Taiwan, entitling patients to full financial coverage for medication. Since many of these orphan drugs are very expensive, hospitals do not provide the drugs without prior reimbursement approval from the Bureau of National Health Insurance. The Bureau requires 4.5 working days to review a patient’s diagnosis report before granting reimbursement for any drugs. In general, it should be noted that Taiwan has been very generous with respect to reimbursement for a variety of rare diseases. Reimbursement levels have been very reasonable and often cover the entire cost of the medication and office visits. There are many patient or parent groups that have successfully lobbied the Taiwanese government for such monies. The various benefits in different countries are illustrated in Table 4.

**Table 4:** Comparison of the various policies on orphan drugs worldwide

Parameters	USA	Japan	Australia	EU
**Legal framework**	Orphan Drug Act (1983)	Orphan Drug Regulation (1993)	Orphan Drug Policy (1998)	Regulation (CE) N°141/2000 (2000)
Administrative authorities involved	FDA /OOPD	MHLW/OPSR (Orphan Drug Division)	TGA	EMEA/COMP
Prevalence of the disease (per 10,000 individuals), justifying the orphan status	7.5	4	1.1	5
Estimation of the population affected, prevalence rate (per 10,000 individuals)	20 millions	No information	No information	25–30 millions
Marketing exclusivity	7 years	10 years	5 years (similar to other drugs)	10 years
Tax credit	Yes: 50% for clinical studies	Yes: 6% for any type of study + limited to 10% of the company’s corporation tax	No	Managed by the member states
Grants for research	Programs of NIH and others	Governmental funds	No	“FP6” + national measures
Reconsideration of applications for orphan designation	No	Yes	Yes (every 12 months)	Yes (every 6 years)
Technical assistance for elaboration of the application file	Yes	Yes	No	Yes
Accelerated marketing procedure	Yes	Yes	Yes	Yes (via the centralized procedure)

Sources: European Parliament 1999 – Stoa publications – Orphan Drugs – PE 167 780/Fin.St. Presentation of Prof Josep Torrent-Farnell (president of the COMP) at the “Annual EuroMeeting 2001”, Barcelona, 6-9 mars 2001. TGA, therapeutic good administration; EMEA, European Agency for the Evaluation of Medicinal Products; COMP, committee for orphan medicinal products; NIH, National Health Institute.

In South Africa, the South African Foundation for Rare Disorders (SAFRD) was established for the first time in South Africa to help support those affected by rare disorders. The SAFRD was established with the assistance of Genzyme Biopharmaceuticals South Africa at the end of 2008. The main goal of the SAFRD is to create a more positive life for those living with a rare disorder by giving the necessary support in as many aspects of living with their disorder as possible. For those affected by rare disorders, the sense of loneliness and lack of understanding from the greater community can sometimes be as overwhelming as the disease itself. The SAFRD is creating a network of those who truly understand and can provide real help to those affected by certain rare disorders. They can share their experiences of living with a rare disorder, thereby helping them to expand their knowledge and understanding of their disease. Some of the goals of the SAFRD include lobbying for the rights of patients and access to therapy, providing patients with access to up to date information, emotional support, and possibly financial assistance. The SAFRD also strives to foster a better understanding of rare disorders among communities throughout South Africa through public relations, awareness campaigns, and fundraising. The SAFRD also endeavors to interact with doctors to improve their knowledge of the conditions and maximize patient outcomes, and with other international organizations, to share the newest information on medical findings and newly developed treatments.[22]

## Challenges in Access to and Affordability of Medicines for Rare Diseases

Despite the progress, no effective and safe treatment is available for many rare diseases. Furthermore, when treatments are available, obstacles are encountered that hinder access and use of these drugs.[23–26]

### Challenges in assessing clinical relevance and cost effectiveness

The methodology for evaluating orphan drug treatments is often still in an experimental phase, hampering positioning in clinical practice.

### Lack of knowledge and training

For many rare diseases, available information is inadequate. Health professionals are often deficient in appropriate training and awareness to be able to diagnose and adequately treat these diseases.

### Deficient diagnostic systems

For many diseases, no diagnostic methods exist, or diagnostic facilities are unavailable. In these cases, diagnosis may be problematic. Consequently, validity, coding, and reproducibility are problems. Although the pace of gene discovery for rare genetic diseases has accelerated during the past decade, in part, due to the success of the Human Genome Project, translation of these discoveries to clinical utility still lags behind.[27]

### High prices

Prices of orphan drugs per treatment episode can be very high. For example, the cost of treatment with enzyme replacement therapies may reach more than US$150,000 per treatment year. The affordability of orphan drugs has become a major issue for payers and is thus a strong driver of tensions between the different stakeholders. Some companies have responded to this by developing programs to facilitate access to orphan drugs. These obstacles to treating rare diseases with orphan drugs exemplify and mirror the global debate of deficiencies in bringing new drugs to patients who need them. Furthermore, advances in pharmacogenomics may lead to treatments benefiting a small subgroup of patients.[28] Whatever the outcome, it seems inevitable that with an increasing number of drugs specifically indicated, and effective, for rare diseases, these medicines will feature more often on future public health agendas. The orphan drugs and few market players are illustrated in the Table 5.

**Table 5:** Orphan drugs and market players

Drug[7]	Company	Therapeutic indication
Zavesca (miglustat)	Actelion Pharmaceuticals US, Inc	Type 1 Gaucher disease
Ventavis (iloprost)	Actelion Pharmaceuticals US, Inc	Pulmonary arterial hypertension (WHO Group I) in patients with NYHA Class III or IV symptoms.
Trisenox (arsenic trioxide injection)	Cephalon, Inc.	Acute promyelocytic leukemia (APL)
Tracleer (bosentan)	Actelion Pharmaceuticals US, Inc	Pulmonary arterial hypertension (WHO Group I) in patients with WHO Class II–IV symptoms
Somavert (pegvisomant for injection)	Pfizer Limited	Acromegaly
Replagal (agalsidase alfa)	Transkaryotic Therapies, Inc.	Fabry’s disease (alpha-galactosidase A deficiency)
Onsenal	Pfizer	Reduction of the number of adenomatous intestinal polyps in familial adenomatous polyposis (FAP)
PhotoBarr (porfimer sodium)	Axcan Pharma International	High-grade dysplasia (HGD)
Litak (cladribine)	Lipomed	Hairy cell leukemia
Glivec (imatinib mesylate)	Novartis	Philadelphia chromosome positive chronic myeloid leukemia in blast crisis, accelerated phase, or in chronic phase after failure of interferon-alpha therapy
Fabrazyme (agalsidase beta)	Genzyme Europe	Fabry disease
Carbaglu (carglumic acid)	Orphan Europe S.A.R.L.	Hyperammonemia
Busilvex (busulfan)	Pierre Fabre Médicament	Allogeneic hematopoietic progenitor cell transplantation for chronic myelogenous leukemia
Aldurazyme (laronidase)	Genzyme Ltd.	Enzyme replacement therapy in patients with a confirmed diagnosis of mucopolysaccharidosis I (MPS I; ±-L-iduronidase deficiency) to treat the non-neurological manifestations of the disease

## Recent Developments in Regulating ‘Orphan Drug’ Approval

The US Food and Drug Administration (FDA) and the European Medicines Agency (EMA) have announced a more streamlined process to help regulators better identity and share information throughout the development process of orphan drug and biologic products, which are developed specifically to treat rare medical conditions. Both agencies have agreed to accept the submission of a single annual report from sponsors of orphan drug and biological products designated by both the US and the EU.

Currently, if an orphan product was granted designation on the exact same day in both the US and EU, the sponsors must submit separate reports to their respective regulatory agency. The use of one annual report will also benefit sponsors by eliminating the duplication of efforts and by simplifying the process that meets the annual reporting requirements of both the US and the EU for orphan designated products.

The single annual report, much like separate agency reports, will provide information to both agencies on the development of orphan medical products, including a review and status of ongoing clinical studies, a description of the investigation plan for the coming year and anticipated or current problems in the process that may impact their designation as an orphan product. The single annual report submission to both regulatory agencies is voluntary and will apply only to sponsors who have obtained an orphan designation status for their product from both the FDA and EMA.[29] Top players in the manufacture of orphan drugs and pipeline drugs in various pharmacological categories are illustrated in Tables 6 and 7.[30,31]

**Table 6:** Companies involved in the manufacture of orphan drugs

Big pharma and established biotech
Pfizer
GlaxoSmithKline
Novartis
Sanofi-Aventis
Roche
Johnson and Johnson
Merck and Co
Eli Lilly
Bayer
Orphan drug specialists
Genzyme
Actelion

**Table 7:** The orphan drug pipeline

Brand name	Generic name
Oncology	
Istodax	Romidepsin
Yondelis	Trabectedin
Omapro	Omacetaxine
Clolar	Clofarabine
Onrigin	Laromustine
TM601	
EGEN – 001	
Central nervous system	
Zenas	Amifampridine
ITI111	Midazolam
H P Acthar Gel	
Respiratory and pulmonary systems	
Surfaxin	
Anti-infectives	
Cayston	Aztreonam lysine
ABthrax	Raxibacumab
Autoimmune and inflammation	
EN 101	
Genetic diseases and dysmorphic syndromes	
Uplyso	Taliglucerase alfa

## Orphan Drugs Versus Essential Medicines

Although the fields of essential medicines and orphan drugs share principles of social justice and equity, Table 8 lists some important ways in which the two groups of medicines differ.[32]

**Table 8:** A comparison of essential medicines and orphan drugs

Aspect	Essential medicines	Orphan drugs
Concrete policies in place since	1977 worldwide	1983 in USA, 2000 in EU
Primary focus	Public health: bringing effective medicines to as many patients as possible	Individual patient: even a single patient warrants all possible treatment
Initiated and developed by	WHO, and Member States	Governments of Australia, EU, Japan and USA; patient groups
Criteria	Drug driven (i.e., drug to be listed on EML is efficacious, safe, cost effective, based on evidence based data, etc.)	Disease driven (i.e., disease to be classified as an orphan drug has low prevalence <5–7.5: 10,000, is life-threatening, etc.)
Policies aim to	Provide established medicines to patients	Provide new medicines to as yet untreatable patients
Target populations	Initially low-income countries, now all countries	High-income countries, developed countries
Economics	Cost-effectiveness, sustainable, and affordable access	Relatively high prices per individual patient, cost-maximization per population

USA, United States of America; EU, European Union; EML, Model of Essential Medicines.

**Figure 1:**
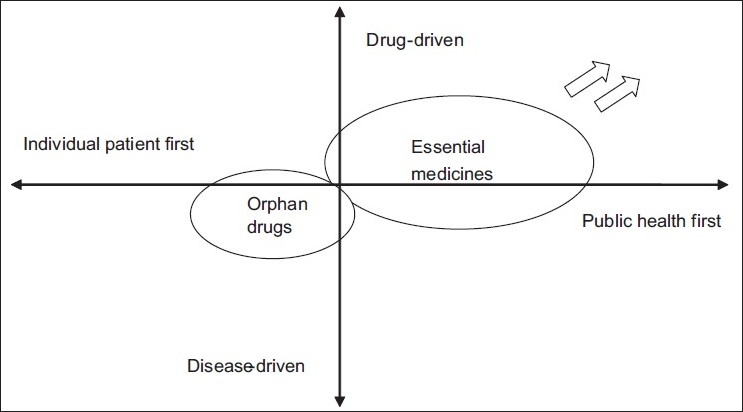
Priorities in bringing important drugs to patients: two dimensions. In this figure, “drug-driven” refers to more emphasis on the drug compound for decision-making (e.g., cost-effectiveness, evidence base). “Disease-driven” refers to more emphasis on the characteristics of the disease-making process. The arrows indicate a future trend based on recent developments

Developments in policies affecting the model of essential medicines (EML) may result in these fields becoming more and more distinct in the future. The primary focus in the orphan drug arena is the individual patient, irrespective of the demands of society at large. This contrasts with the more “utilitarian” public health approach of the current EML definitions. Moreover, the two systems also differ in their drug/disease orientation. Figure 1 captures these two dimensions. The domain of the EML is dominated by public health concerns (i.e., priority diseases) and proven effectiveness of medicines through the methods of “evidence-based medicine.” The 2002 revisions of the EML entry criteria show an increased move toward the upper right quadrant. Therefore, if current EML definitions are applied strictly, both fields may “lose touch.” This is an unwanted situation in the future developments in the pharmaceutical field.

## Market Exclusivity

Orphan drug exclusivity applies to those vaccines and diagnostic or preventive drugs either designed to affect conditions that afflict a relatively small number of people or for which there is no reasonable expectation of the recovery of research and development costs.[33]

The approval of an application for orphan designation is based upon the information submitted by the sponsor. A drug that has obtained orphan designation is said to have “orphan status.”[25] Sponsors need to follow the “standard regulatory requirements and process for obtaining market approval.”[34]

A sponsor may request orphan drug designation for a previously unapproved drug or for an already marketed drug. More than one sponsor may receive orphan drug designation for the same drug for the same rare disease or condition. Drug with orphan status enjoys exclusive approval and market exclusivity. Table 9 gives us an overview of market exclusivity for orphan drugs for different countries.

**Table 9:** Orphan drug market exclusivity

Countries	Market exclusivity
USA	7 Years
Europe	10 Years
Japan	10 Years
Korea	6 Years
Singapore	10 Years
Taiwan	10 Years

## The Future of ‘Orphan Drug’: An International Dimension

As the Asian pharmaceutical markets grow, so too will opportunities for orphan drugs in Asia. Asian governments are becoming more aware of the importance of orphan drugs, and reimbursements for these products will increase in the future. Each Asian market, however, is different, and one must study each Asian country’s orphan drug regulations and markets to be successful.

Rare diseases need more attention due to lack of proper diagnosis and treatment. Treatment and prevention for rare diseases is considered as “no man’s land.” The EU parliament should provide more benefits like tax incentive, special status, and reimbursement for these orphan drugs. This encouragement can bring in a revolution among the pharmaceutical and biotechnology companies for developing and marketing orphan drugs. EURODIS and other organizations are creating awareness on rare diseases and are also influencing governments in bringing legislation acts for better quality of life for these special people. EURORDIS and National Alliances have announced “rare day” on February 29th and dedicated this day to special people who are affected by rare diseases. Henceforth, 29th February will be called “the rare disease day.”[35]

The future of the orphan drug industry will depend heavily upon the entry of biogenerics, since biologics account for over 50% of the orphan drug market. It can be expected that the orphan drug market growth will remain positive as more and more governments are taking action to promote this sector, especially in Asia.

Australia, Japan, Singapore, Taiwan, and Korea have already implemented legislation for promoting research on orphan drugs, India and New Zealand are in the process of establishing similar regulatory processes. With more countries adopting similar legislation, it can be expected that market potential will increase.

Pharmacogenomics is forcing a paradigm shift in patient care. An inevitable impact will be shrinking patient populations as this approach may eventually lead to a scenario where the majority of blockbuster drugs are suitable only for a small group of patients, the number of which may very well fall within the definition of rare diseases. Such developments could lead to a redefinition of the limits set by the ODA.

The issue of orphan drugs becomes more important for third world countries like India, which are affected the most. Why no new drug is coming up or nobody is investing in research and development (R and D) in malaria, leishmania, etc. Usually we criticize the pharmaceutical industry or manufacturers for this. This is a controversial issue. The manufacturers cannot be entirely blamed for this. It is not easy to produce and market orphan drugs. The manufacturers of drugs have to amortize their operational expenses, their research investment, and they have to make reasonable profit so that they can finance new ventures in the future. It is calculated that return on investment for the average new chemical entity (NCE) is barely 6-8%, a figure with serious implications for a prudent businessman. One area where the ODA has not provided very strong incentives is new drugs and vaccines for the neglected diseases such as malaria and tuberculosis that affect poor countries. Given their low prevalence in the United States, diseases that predominantly affect poor countries are technically eligible for all of the incentive provisions of the ODA. However, developing countries lack the resources to afford these drugs, with many devoting as little as $2 per capita per year to heath care. Also R and D costs have been rising and drug prices declining. Hence these figures too suggest a flow from less attractive to more attractive alternatives for investment.

A country should try to produce important drugs for the benefit of the whole world, depending on the R and D investment, the return on such investment, the tax and patent incentives, and its regulatory policies. Agreement of these points might lead to beneficial changes in our national thinking and prevent “orphanization of new drugs.”

## References

[1] Brewer GJ (2009). Drug development for orphan diseases in the context of personalized medicine. Transl Res.

[2] Villa S, Compagni A, Reich MR (2009). Orphan drug legislation: Lessons for neglected tropical diseases. Int J Health Plann Manage.

[3] Cote T, Kelkar A, Xu K, Braun MM, Phillips MI (2010). Orphan products: An emerging trend in drug approvals. Nat Rev Drug Discov.

[4] Thielke D, Thyssen JP, Hansen BJ (2006). Orphan drugs--medications for patients with rare diseases. Ugeskr Laeger.

[5] http://www.orpha.net/consor/cgi-bin/Education_AboutOrphanDrugs.php?lng=EN.

[6] Ariyanchira S Global Markets for Orphan Drugs, BCC Research, BCC00191, PHM038B, January 2007. http://www.bccresearch.com/report/PHM038B.html.

[7] Ariyanchira S Global Markets for Orphan Drugs, BCC Research, BCC00191, PHM038C, May 2010. http://www.bccresearch.com/report/PHM038C.html.

[8] http://www.fda.gov/.

[9] (1992). United States Food and Drug Administration. The Orphan Drug Regulations. Final rule, 57 FR 62076 21 CFR Part 316.

[10] United States Congress United States Congress. Rare diseases act of 2002. Public Law 107–80.

[11] (2001). Office of Inspector General. United States Department of Health and Human Services. The Orphan Drug Act implementation and impact.

[12] http://ec.europa.eu/research/fp7/index_en.cfm.

[13] http://www.ema.europa.eu/pdfs/human/comp/29007207en.pdf.

[14] Shah RR Regulatory framework for the treatment of orphan diseases. http://www.ncbi.nlm.nih.gov/bookshelf/br.fcgi?book=fabryandpart=A745.

[15] Sansom L (2005). Evaluation and subsidy of orphan drugs in Australia. Health Technology Assessment International. Meeting. Ital J Public Health.

[16] http://www.gopharmaceutical.com/orphanp.html.

[17] http://www.pharmainfo.net/reviews/orphan-diseases-indian-perspective.

[18] http://www.kfda.go.kr.

[19] http://www.pacificbridgemedical.com/publications/asia/2006_orphan_drugs_in_asia.

[20] http://www.ncbi.nlm.nih.gov/pmc/articles/PMC2631478/.

[21] Asbury CH (1991). The Orphan Drug Act. The first 7-years. JAMA.

[22] http://www.safrd.co.za/.

[23] http://www.fda.gov/ForIndustry/DevelopingProductsforRareDiseasesConditions/default.htm.

[24] http://www.who.int/inf-pr-1999/en/pr99-74.html.

[25] Stolk P, Willemen MJ, Leufkens HG (2006). Rare essentials: Drugs for rare diseases as essential medicines. Bull World Health Organ.

[26] Brewer GJ (2006). Fundamental problems lie ahead in the drug discovery and commercialization process: Restructuring of the pharmaceutical industry and an improved partnership with academia are required. J Investig Med.

[27] Das S, Bale SJ, Ledbetter DH (2008). Molecular genetic testing for ultra rare diseases: Models for translation from the research laboratory to the CLIA-certified diagnostic laboratory. Genet Med.

[28] Scheindlin S (2006). Rare diseases, orphan drugs, and orphaned patients. Mole Interv,.

[29] (2010). Biospectrum Asia Edition. http://www.cybermediaservicesdigitalmag.com/admin/magazines/vol5_issue6/digimag.html.

[30] (2010). Opportunities in Orphan Drugs: Strategies for developing maximum returns from niche indications, Business Insights. http://www.globalbusinessinsights.com/content/rbhc0251m.pdf.

[31] http://www.fda.gov/NewsEvents/Newsroom/PressAnnouncements/ucm202300.htm.

[32] http://www.who.int/bulletin/volumes/84/9/06-031518ab/en/index.html.

[33] (1992). FDA. Final Rule, 21 CFR 316. Orphan Drug Regulations 57 FR 62076 December 29. http://www.fda.gov/orphan/designat/apply.htm.

[34] OOPD Program Overview. http://www.fda.gov/orphan/progovw.htm.

[35] Fischer A, Borensztein P, Roussel C (2005). The European rare diseases therapeutic initiative. PLoS Med.

